# Development of RIKEN Plant Metabolome MetaDatabase

**DOI:** 10.1093/pcp/pcab173

**Published:** 2021-12-17

**Authors:** Atsushi Fukushima, Mikiko Takahashi, Hideki Nagasaki, Yusuke Aono, Makoto Kobayashi, Miyako Kusano, Kazuki Saito, Norio Kobayashi, Masanori Arita

**Affiliations:** Metabolome Informatics Research Team, RIKEN Center for Sustainable Resource Science, 1-7-22 Suehiro, Tsurumi, Yokohama, Kanagawa 230-0045, Japan; Metabolome Informatics Research Team, RIKEN Center for Sustainable Resource Science, 1-7-22 Suehiro, Tsurumi, Yokohama, Kanagawa 230-0045, Japan; Metabolome Informatics Research Team, RIKEN Center for Sustainable Resource Science, 1-7-22 Suehiro, Tsurumi, Yokohama, Kanagawa 230-0045, Japan; Degree Programs in Life and Earth Sciences, University of Tsukuba, 1-1-1 Tennodai, Tsukuba, Ibaraki 305-8572, Japan; Metabolome Informatics Research Team, RIKEN Center for Sustainable Resource Science, 1-7-22 Suehiro, Tsurumi, Yokohama, Kanagawa 230-0045, Japan; Metabolome Informatics Research Team, RIKEN Center for Sustainable Resource Science, 1-7-22 Suehiro, Tsurumi, Yokohama, Kanagawa 230-0045, Japan; Faculty of Life and Environmental Science, University of Tsukuba, 1-1-1 Tennodai, Tsukuba, Ibaraki 305-8572, Japan; Tsukuba Plant Innovation Research Center, University of Tsukuba, 1-1-1 Tennodai, Tsukuba, Ibaraki 305-8572, Japan; Metabolome Informatics Research Team, RIKEN Center for Sustainable Resource Science, 1-7-22 Suehiro, Tsurumi, Yokohama, Kanagawa 230-0045, Japan; Metabolome Informatics Research Team, RIKEN Center for Sustainable Resource Science, 1-7-22 Suehiro, Tsurumi, Yokohama, Kanagawa 230-0045, Japan; Data Knowledge Organization Unit, RIKEN Information R&D and Strategy Headquarters, 2-1 Hirosawa, Wako, Saitama 351-0198, Japan; Metabolome Informatics Research Team, RIKEN Center for Sustainable Resource Science, 1-7-22 Suehiro, Tsurumi, Yokohama, Kanagawa 230-0045, Japan; Bioinformation and DDBJ Center, National Institute of Genetics, Yata 1111, Mishima, Shizuoka 411-8540, Japan

**Keywords:** Data sharing, Metabolite profiling, Metabolomics, Plant metabolism, Semantic web

## Abstract

The advancement of metabolomics in terms of techniques for measuring small molecules has enabled the rapid detection and quantification of numerous cellular metabolites. Metabolomic data provide new opportunities to gain a deeper understanding of plant metabolism that can improve the health of both plants and humans that consume them. Although major public repositories for general metabolomic data have been established, the community still has shortcomings related to data sharing, especially in terms of data reanalysis, reusability and reproducibility. To address these issues, we developed the RIKEN Plant Metabolome MetaDatabase (RIKEN PMM, http://metabobank.riken.jp/pmm/db/plantMetabolomics), which stores mass spectrometry-based (e.g. gas chromatography–MS-based) metabolite profiling data of plants together with their detailed, structured experimental metadata, including sampling and experimental procedures. Our metadata are described as Linked Open Data based on the Resource Description Framework using standardized and controlled vocabularies, such as the Metabolomics Standards Initiative Ontology, which are to be integrated with various life and biomedical science data using the World Wide Web. RIKEN PMM implements intuitive and interactive operations for plant metabolome data, including raw data (netCDF format), mass spectra (NIST MSP format) and metabolite annotations. The feature is suitable not only for biologists who are interested in metabolomic phenotypes, but also for researchers who would like to investigate life science in general through plant metabolomic approaches.

## Introduction

The metabolome is the whole set of low-molecular-weight metabolites (<1,500 Da) within a cell (Oliver et al. 1998). Recent analytical platforms, such as mass spectrometry (MS) and nuclear magnetic resonance spectroscopy, have enabled simultaneous measurement of the steady-state levels of metabolites. The growing methodologies and fields are called metabolomics. The literature suggests that the number of metabolites produced in the plant kingdom exceeds 100,000 (Fiehn 2002, Dixon and Strack 2003, Afendi et al. 2012). For example, it is estimated that the model plant *Arabidopsis thaliana* produces approximately 5,000 metabolites (Fernie et al. 2004, Saito and Matsuda 2010). Given that plants and crops serve as rich resources for food and drug development (Putri et al. 2013, Rai and Saito 2016), metabolomic data sharing and standardization are extremely important to reuse and reanalyze such data and to increase transparency of the study process.

A number of databases containing information related to metabolome analysis have been established in recent decades (Horai et al. 2010, Fukushima and Kusano 2013, Gurevich et al. 2018, Wishart et al. 2018, 2020). Since the establishment of the Metabolomics Standards Initiative (MSI), substantial efforts have been made in the field to overcome challenges related to the appropriate reporting of metabolomics studies (Fiehn et al. 2007a). As general-purpose metabolomics data repositories, two major databases have been established: MetaboLights (Haug et al. 2020) and Metabolomics Workbench (Sud et al. 2016). MetabolomeXchange (http://www.metabolomexchange.org/) indicates that, as of 1 July 2021, more than 800 metabolomic datasets are publicly available in MetaboLights and more than 1,400 in Metabolomics Workbench. Nevertheless, Spicer et al. (2017a) revealed that, based on an analysis of publicly available metabolomics (meta-)data, the majority of the shared metadata have substantial limitations.

Semantic web technologies facilitate the provision of Findable, Accessible, Interoperable and Reusable data (Wilkinson et al. 2016) on the World Wide Web (WWW). Of these technologies, the keys are Resource Description Framework (RDF) and SPARQL Protocol and RDF Query Language (SPARQL), which are global standards formulated by the WWW Consortium. These are powerful tools for realizing low-cost metadata management and the integration of distributed global data including omics data. Examples include KEGG/GenomeNet LinkDB RDF (Kanehisa et al. 2021) and UniProt RDF in genomics (UniProt 2019), Expression Atlas (Papatheodorou et al. 2020) and RefEx (Ono et al. 2017) in transcriptomics, jPOST in proteomics (Watanabe et al. 2021b), glycoPOST in glycomics (Yamada et al. 2020, Watanabe et al. 2021a), PubChem RDF in chemical information (Fu et al. 2015), SPARQLing biochemical reaction data (Rhea) in biochemical information (Lombardot et al. 2019), and RIKEN MetaDatabase (http://metadb.riken.jp) for a wide range of healthcare and life sciences (Kobayashi et al. 2018).

Although major public repositories for general metabolomic data have been launched, the metabolomics community still needs to address the shortcomings of data sharing, especially in terms of reanalysis, reusability and reproducibility of data. In this study, we have developed the RIKEN Plant Metabolome MetaDatabase (RIKEN PMM, http://metabobank.riken.jp/pmm/db/plantMetabolomics), which mainly provides MS-based metabolite profiling data of plants together with their detailed and structured metadata as Linked Open Data (LOD) based on the semantic web. To introduce our reanalysis approach with RIKEN PMM, we have shared our gas chromatography (GC)–MS data reanalysis workflow.

## Results

### Database overview and content

**Fig. 1:**
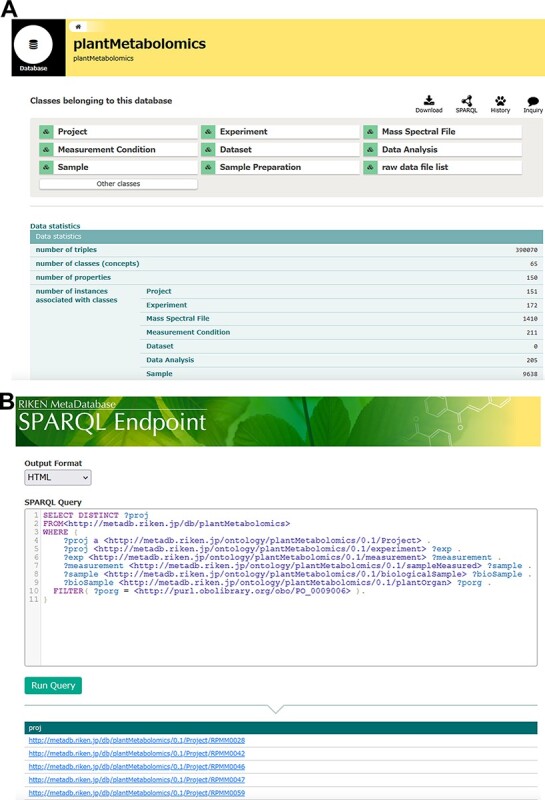
Specialized views in RIKEN PMM. (A) Database view shows the data statistics, including the number of classes like Project, Experiment and Dataset, and other statistical data. (B) SPARQL search view allows us to input a SPARQL query and returns query results in various formats such as HTML and a spreadsheet style. The example query can retrieve all of the projects related to plant ontology ‘shoot system’ (PO:0009006).

The RIKEN PMM is implemented on top of the RIKEN MetaDatabase, which provides a biologist-friendly graphical user interface, including tabular and card forms that are familiar to biologists to show classes and instances of graph-based RDF datasets simultaneously as well as SPARQL endpoint functions as the application programming interface (**Fig. 1**). Briefly, the key features are as follows:

1. Unlike a typical relational database, it is easy to extend and revise data schema due to RDF.
2. RIKEN PMM promotes the distribution of plant metabolome data as LOD and efficient data integration across scattered databases.

In the current database, we archived public plant metabolome datasets containing a total of 151 projects (**Supplementary Table S1**), encompassing >9K biological samples and >8.6K raw data files (**Fig. 1**). It also spans over 40 different plant species including Arabidopsis, rice, tomato, soybean and lettuce (**Supplementary Material 1**). We compared the share of samples from Plantae in major metabolome databases. The total number of Plantae samples in the RIKEN PMM was 8,809 (by 13 July 2021). However, the shares of species included 42% Brassicales, 26% Poales and 15% Solanales. This suggests that there is no major bias. In the case of MetaboLights, the total number of samples was 49,496 (as of 13 July 2021), but 65% of them were from studies on the Lolium family (Subbaraj et al. 2019). The total number of Plantae samples was 28 (as of 13 July 2021) in Metabolomics Workbench. Thus, compared with other databases, our datasets were well balanced in terms of samples from specific plants.

We also opened almost all of the datasets in open standard file formats, including netCDF and Analysis Base Framework (ABF). The latter ABF file was converted using the freely available AbfConverter (http://www.reifycs.com/AbfConverter/index.html). Users can access all raw metabolite profile data files, such as those in the netCDF format, at least for GC–MS-based studies from RIKEN but with the exception of metadata from Kazusa DNA Research Institute (https://www.kazusa.or.jp/). As an example, you can examine such files at the following URL: http://metabobank.riken.jp/pmm/db/plantMetabolomics/http://metadb.riken.jp/db/plantMetabolomics/0.1/RawDataSet/RPMM0026_root_02_Polar_1.

A raw data file in netCDF format can be accessed at http://metabobank.riken.jp/data/RPMM0026/PolarMetabolites/RawDataset/root_02_Polar_1.cdf. This file is associated with the accession number RPMM0026 (Ichihashi et al. 2018) and corresponds to a root sample.

To facilitate the sharing of our experimental design (called phenodata), metabolite annotation and data matrix processed in each project, we also provide them as simple text and/or in CSV file format. Among 151 datasets, 90 datasets contain plant samples from Kazusa DNA Research Institute, linking the raw data with MassBase (Ara et al. 2021). Users can distinguish these sample names with the prefixes ‘RPMM’ and ‘MN’ (Metabolonote) (Ara et al. 2015). Excluding Kazusa’s dataset (MN#####), only three datasets from RIKEN (RPMM####), i.e. RPMM0001, RPMM0002 and RPMM0006, contain direct URL links to MetaboLights. This is due to sharing the three datasets and their metadata in RIKEN PMM, MetaboLights and Metabolonote.

### Implementation and design

We have developed a novel ontology, called the Plant Metabolomics Ontology, to describe our metabolome data (https://github.com/afukushima/rpmm-metadata). This ontology takes over the DNA Data Bank of Japan (DDBJ) data structure (Ogasawara et al. 2020), including the BioSample database, and is extended with additional classes, such as experimental condition and data analysis. These additional classes describe the concepts of MS-based metabolomics (e.g. GC–MS) and statistical data analysis. The metabolomic part of the Plant Metabolomics Ontology is designed to realize metadata interoperability according to the recommendation of the MSI (http://www.metabolomics-msi.org/) (Fiehn et al. 2007a, 2007b) by introducing ontology terms (classes) defined in the Metabolomics Standards Initiative Ontology (MSIO, https://github.com/ISA-tools/MSIO) for example.

### Browsing and searching metabolomic data

**Fig. 2:**
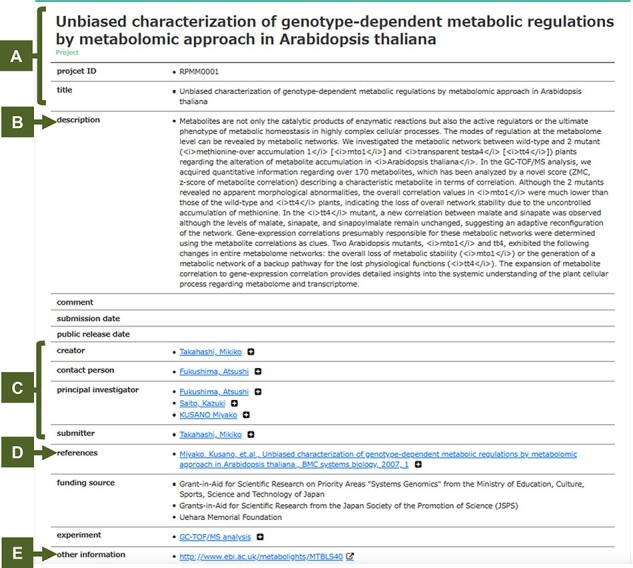
An example of the detailed information of Arabidopsis genotype-dependent metabolome data. (A) represents the project’s title and its unique identifier, while (B) explains this project, including a description of the goals and aims of this study. Typically, the abstract from the associated publication was set, (C) contains creator, contact person, principal investigator and submitter names, (D) is the links to the corresponding literature (e.g. PubMed and DOI) and (E) the links to other information and databases. This can be used to view an instance and its triplets linked to other instances or reverse-linked from other instances. A user can walk through an instance linked via a triplet to show further triplets with the selected instance.

**Fig. 3:**
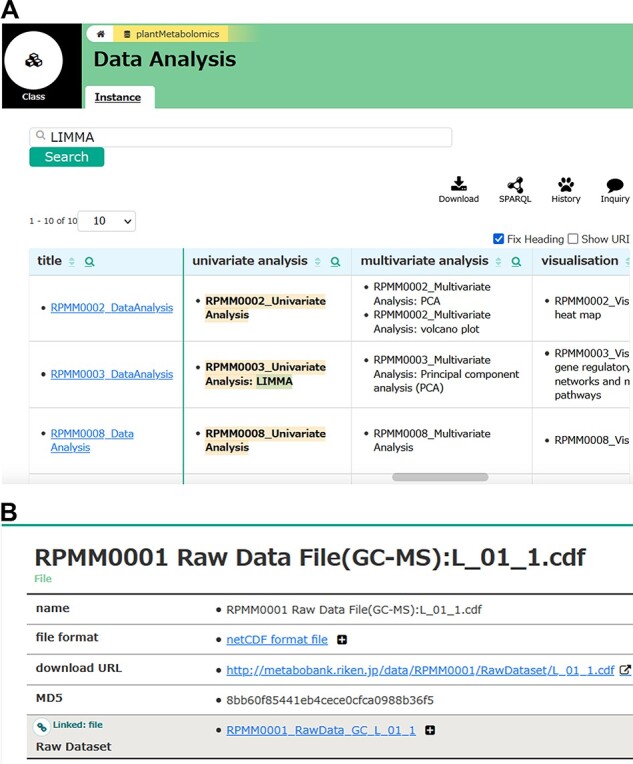
An example of search and download functions in RIKEN PMM. (A) A user can retrieve related projects with the univariate analysis method ‘LIMMA’ as a keyword query. LIMMA represents ‘Linear Models for Microarray Data’ (Ritchie et al. 2015). (B) Users can also access the raw data of each bio sample (e.g. L_01_1.cdf) in RPMM0001 (Kusano et al. 2007).

Users can browse our projects in RIKEN PMM, providing a set of all of the public studies currently available. Almost all studies have been reported in peer-reviewed journals. **Fig. 2** shows an example of the detailed information of genotype-dependent metabolome data in *A. thaliana* leaves (Kusano et al. 2007). The page contains the project’s title, its unique identifier, description of the project, information about contributors to the project, links to the corresponding publication, and links to other information and databases. A user can walk through an instance linked via a triplet to show further triplets with the selected instance. RIKEN PMM provides a user-friendly web interface to extract metabolome-related information such as ‘Sample’, ‘Experiment’, ‘Measurement’ and ‘Data Analysis’. **Fig. 3** shows an example of the search and download functions in RIKEN PMM. For example, users can retrieve related projects with the univariate analysis method ‘LIMMA’ as a keyword query (**Fig. 3A**). Users can also download the raw data of each biosample in each project (**Fig. 3B**).

### Access, privacy policy and license

All of the data in RIKEN PMM are available under a CC-BY-4.0 license as open data, which grants free access and reuse of our public data. We also developed the R package ‘rRPMM’ (https://github.com/afukushima/rRPMM) to download and parse our metadata from the RIKEN PMM (**Supplementary Materials 2 and 3**). An accessor for the RIKEN PMM converts the downloaded metadata to an R list. For example, users can visualize the species distribution in our database (**Supplementary Material 1**).

### Sharing our GC–MS-data-reanalysis workflow

To introduce our reanalysis approach, we have shared our GC–MS-data-reanalysis workflow with our ‘rRPMM’ (https://github.com/afukushima/rRPMM) and ‘eRah’ packages (Domingo-Almenara et al. 2016) (**Supplementary Material 4**). This R-based workflow consists of a process to (i) obtain all raw data (e.g. netCDF files) from a Project, (ii) preprocess the data (e.g. peak deconvolution and alignment) and (iii) identify and annotate metabolites using a public mass-spectral library such as GMD (Kopka et al. 2005).

## Discussion

We have developed a semantic web-based metadata database known as the RIKEN PMM that enables efficient sharing, spreading and retrieving of plant metabolome data using native RDF technologies. We have also developed easy-to-use spreadsheet software to rapidly generate RDF data, provided an accessor R package rRPMM and shared our data reanalysis workflow. There are domain-specific metabolome databases that adhere to the guidelines of the MSI (Fiehn et al. 2007a), including MetabolomeExpress (Carroll et al. 2010, 2015), Mery-B (Ferry-Dumazet et al. 2011) and MeKO (Fukushima et al. 2014). An important point is that the minimum reporting standards in plant science are complied with the most (Spicer et al. 2017a). RIKEN PMM also encourages minimum reporting guidelines for metabolome data analysis, including data transformation, scaling and normalization methods with existing and/or new ontologies (Considine and Salek 2019). As suggested by Spicer et al. (2017b) and in agreement with recently proposed reporting standards (Alseekh et al. 2021), it is time to discuss the revision of the MSI guidelines.

Despite the proposal of minimum reporting guidelines for data analysis in metabolomics for over a decade (Goodacre et al. 2007), the need to improve metadata completeness has been under discussion for some time (Considine and Salek 2019). It is also important to develop an efficient e-infrastructure and data analysis workflow for metabolomics, examples of which include XCMSonline (Forsberg et al. 2018), PhenoMeNal (Phenome and Metabolome aNalysis) (Peters et al. 2019), MetabolomeExpress (Carroll et al. 2010, 2015) and WebSpecmine (Cardoso et al. 2019). Other collaborative approaches also exist with Jupyter notebooks (e.g. see https://www.metabolomicsworkbench.org/data/AnalyzeUsingJupyterNotebooks.php) and R markdown files for metabolomics (Considine and Salek 2019, Mendez et al. 2019). Along with enhancing the metadata of metabolomic data analysis, it is preferable to recognize the importance and prospects of good experimental designs and to be aware of problems related to sample size.

**Fig. 4:**
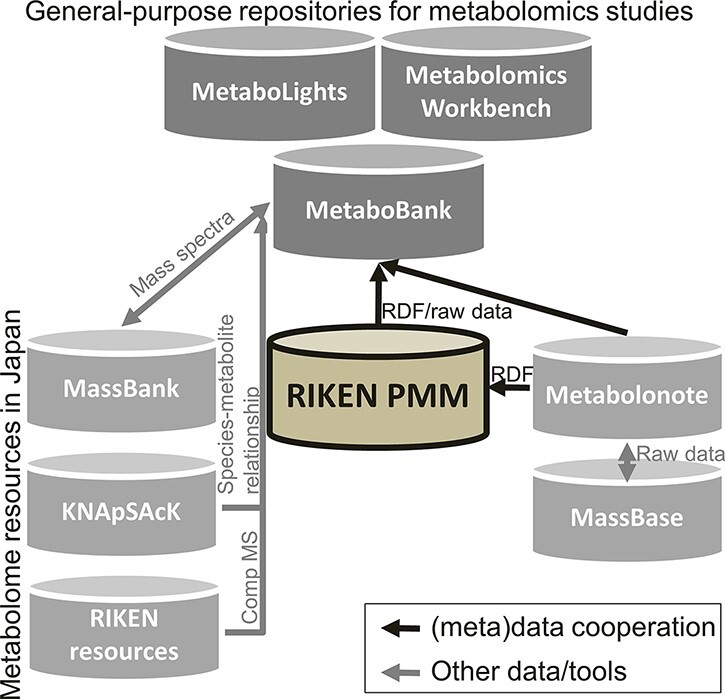
An overview of metabolomics data resources with RIKEN PMM. An open-access general-purpose repository for metabolomics studies across the world including MetaboLights, Metabolomics Workbench and a newly developed database named MetaboBank in DDBJ. Other major metabolome data resources existing in Japan are MassBank, KNApSAcK and other resources related to computational mass spectrometry (Comp MS), such as MS-DIAL in RIKEN (http://prime.psc.riken.jp/compms/index.html). RIKEN PMM was established in 2018 with the aim of providing opportunities to work with and develop applications for plant metabolomics data based on FAIR principles. The upcoming MetaboBank resource will collect and assemble (meta)data from RIKEN and Kazusa DNA Research Institute (KDRI) as initial data. Acceptable (meta)data in MetaboBank include not only mass spectrometry-based data but also all platforms associated with metabolomics studies (e.g. NMR). Other resouces, such as mass spectra data in MassBank, compounds, species–metabolite relationships, and pathway information in KNApSAcK, will contribute to the future development of MetaboBank.

The Plant Metabolomics Ontology is a graph representation of the meaning of terms or concepts in plant metabolomics, which we developed (see also our GitHub page at https://github.com/afukushima/rpmm-metadata). It was implemented as LOD, which aims to progress beyond the conventional Web and provide public accessibility to interlinked datasets from different resources. We, the plant metabolomics researchers at RIKEN, Kazusa DNA Research Institute, and DDBJ, have discussed the domain of plant metabolomics, aims of the ontology and community use case of the ontology. The ontology structure is defined as minimum sets of our data model. Integration of other datasets, including genomics and/or meta-genomics data, now represents an ongoing and worldwide challenge in the field of open life science. We are sure that our work will contribute to future collaborations consisting of different nodes such as MetaboLights, Metabolomics Workbench, MetaboBank in DDBJ and MetabolomeXchange (**Fig. 4**).

In summary, our framework can provide all metadata required for reanalyzing and reusing metabolomic data and will contribute to the development of another general-purpose metabolomics repository, called MetaboBank developed by DDBJ (Ogasawara et al. 2020). Our approach also enables the archiving of data integrated among different disciplines. The data-sharing aspect discussed in this paper will pave the way for discoverable, reproducible and reusable metabolomic data as well as the robust interpretation of plant metabolomic data.

## Supplementary Material

pcab173_Supp

## Data Availability

The raw data underlying this article are available in RIKEN Plant Metabolome MetaDatabase (PMM) (http://metabobank.riken.jp/pmm/db/plantMetabolomics), DropMet (http://prime.psc.riken.jp/menta.cgi/prime/drop_index) and MassBase (http://webs2.kazusa.or.jp/massbase/).
