# Supplementary material for: Development of RIKEN Plant Metabolome MetaDatabase
Source: Plant Cell Physiol. 2021 Dec 17;63(3):433–40. doi: 10.1093/pcp/pcab173 (PMC8917833; doi:10.1093/pcp/pcab173)
Supplement: pcab173_Supp [file pcab173_supp.zip › pcp-2021-e-00297-File006.pdf]

A

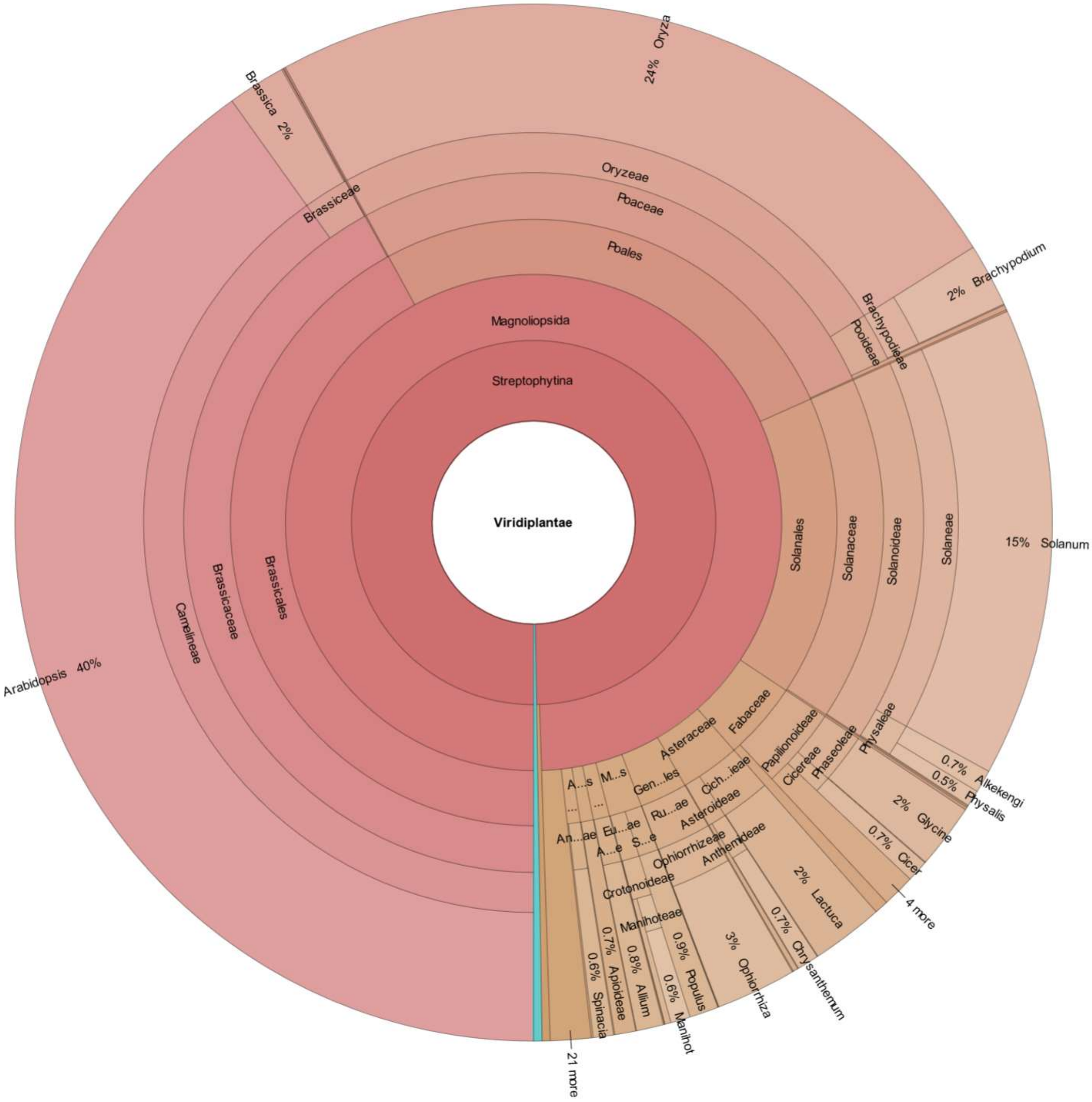

Supplementary material 1. The shares of samples from Plantae in existing metabolome databases. (A) pie chart showing plant species in RIKEN PMM visualized by Krona (Ondov et al. 2011). The data were extracted from the SPARQL endpoint of RIKEN PMM, XML file of MetaboLights, or metabolomicsWorkbenchR (https://github.com/computational-metabolomics/metabolomicsWorkbenchR), and data from multiple species in their datasets were removed.

B

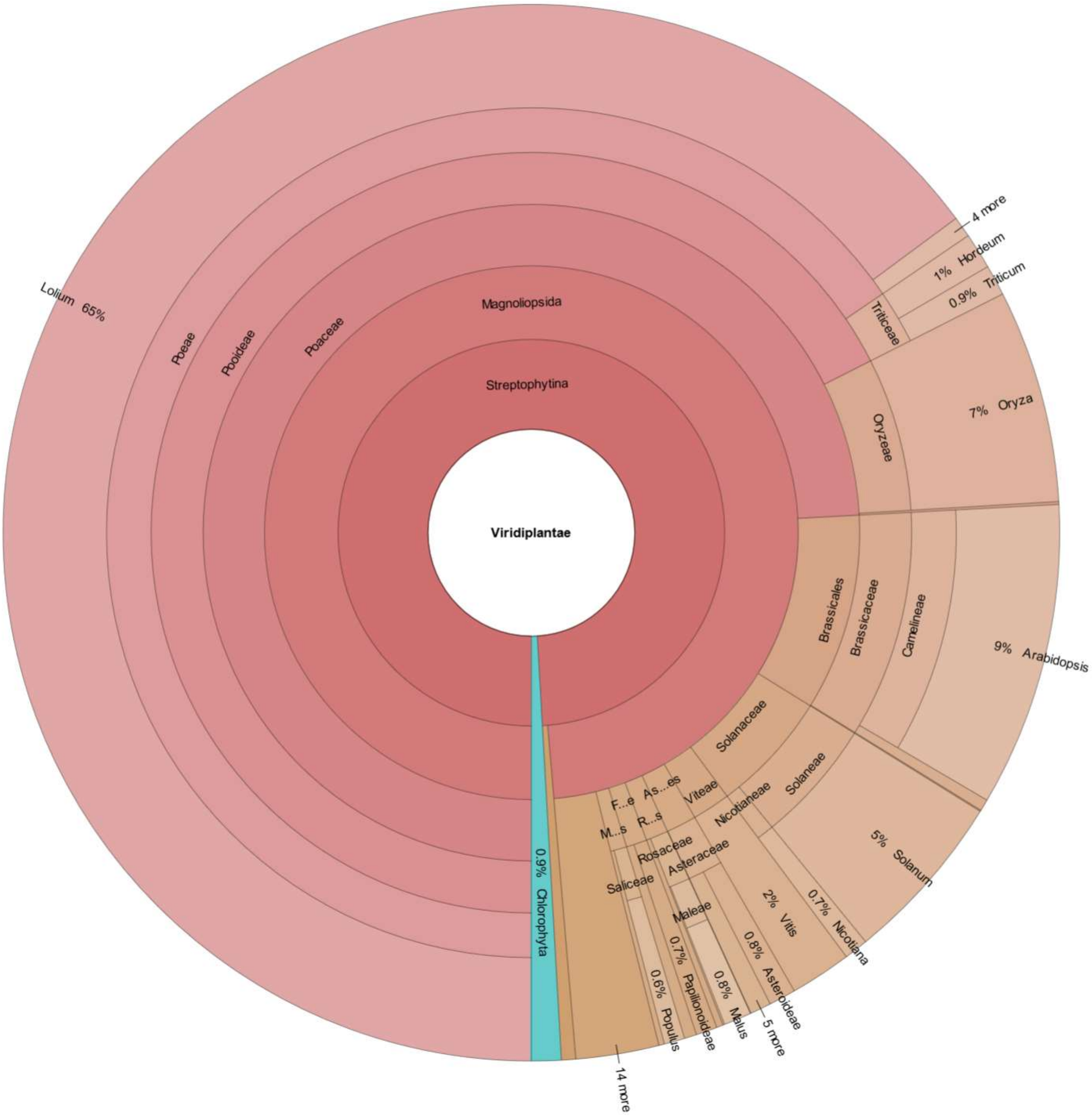

(B) pie chart showing plant species in MetaboLights

C

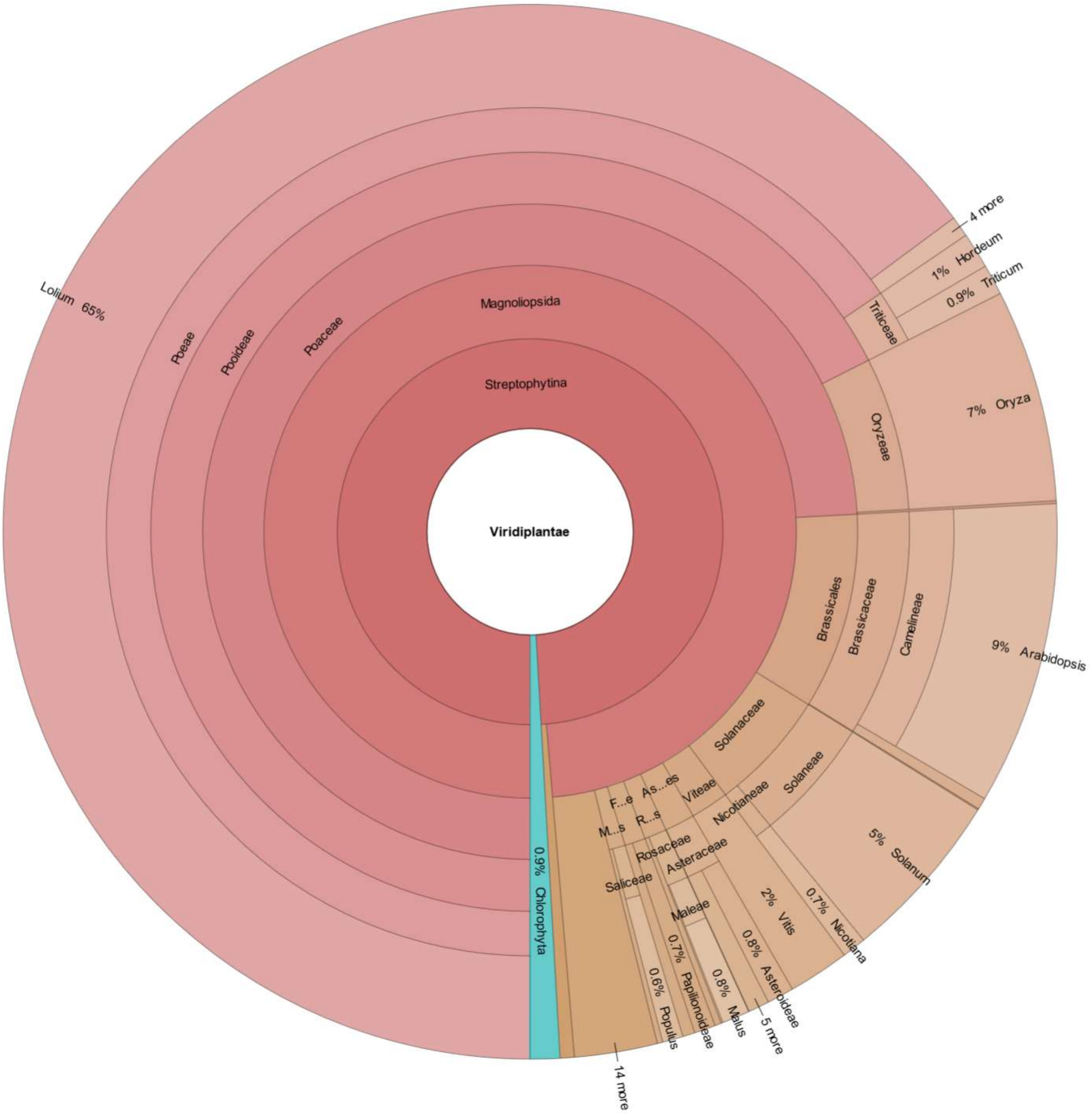

(C) pie chart showing plant species in Metabolomics Workbench
