# Supplementary material for: Development of RIKEN Plant Metabolome MetaDatabase
Source: Plant Cell Physiol. 2021 Dec 17;63(3):433–40. doi: 10.1093/pcp/pcab173 (PMC8917833; doi:10.1093/pcp/pcab173)
Supplement: pcab173_Supp [file pcab173_supp.zip › pcp-2021-e-00297-File007.pdf]

Supplementary material 2. rRPMM's vignette file.

rRPMM - An accessor for RIKEN Plant Metabolome Metadatabase (RPMM)

Atsushi Fukushima1*

1RIKEN CSRS

*afukushima@gmail.com

2021-07-09

Package

rRPMM 0.99.0

Contents

- 1 Introduction
- 2 Installation
- 3 Usage
- 4 Getting all the raw data (netCDF) files in a Project (Accession #: RPMM0001)
  - 4.1 Using ABF* format files (*Reifycs's Analysis Base File)
- Session information

1 Introduction

RIKEN Plant Metabolome MetaDatabase (RPMM) is used to provide integrated plant metabolome data generated by RIKEN. The database provides data in the bases of standardized metadata techniques including semantic web and Resource Description Framework (RDF). The rRPMM package is an accessor for RPMM.

2 Installation

```
# devtools::install_github("afukushima/rRPMM", build_vignettes = TRUE)
```

3 Usage

```
library(SPARQL)
library(rRPMM)
library(plotly)
library(dplyr)
```

```
res <- RPMM_numOfprojects_species()
resMat <- data.frame(gsub("@en", "", res$speciesLabel), res$callret.4)
colnames(resMat) <- c("Species", "num")

## for pie chart with plotly
resMat_1over <- resMat[resMat$num>1, ]
resMat_others <- resMat[resMat$num==1, ]
others <- list("Others", sum(resMat_others$num))
resMatfin <- rbind(resMat_1over, data.frame(Species="others", num="28"))

p <- plot_ly(resMatfin, labels = ~Species, values = ~num, type = 'pie') %>%
     layout(title = 'RPMM projects (Oct 22, 2020)',
          xaxis = list(showgrid = FALSE, zeroline = FALSE,
                      showticklabels = FALSE),
          yaxis = list(showgrid = FALSE, zeroline = FALSE,
                      showticklabels = FALSE))
p
```

4 Getting all the raw data (netCDF) files in a Project (Accession #: RPMM0001)

```
res <- RPMM_get_rawdata_files(project = "RPMM0001")
head(res$url)
## [1]
"¥"http://metabobank.riken.jp/data/RPMM0001/RawDataset/L_01_1.cdf¥"^^<http://ww
w.w3.org/2001/XMLSchema#anyURI>"
## [2]
"¥"http://metabobank.riken.jp/data/RPMM0001/RawDataset/L_02_1.cdf¥"^^<http://ww
w.w3.org/2001/XMLSchema#anyURI>"
## [3]
"¥"http://metabobank.riken.jp/data/RPMM0001/RawDataset/L_03_1.cdf¥"^^<http://ww
w.w3.org/2001/XMLSchema#anyURI>"
## [4]
"¥"http://metabobank.riken.jp/data/RPMM0001/RawDataset/L_04_1.cdf¥"^^<http://ww
w.w3.org/2001/XMLSchema#anyURI>"
## [5]
"¥"http://metabobank.riken.jp/data/RPMM0001/RawDataset/L_05_1.cdf¥"^^<http://ww
w.w3.org/2001/XMLSchema#anyURI>"
## [6]
"¥"http://metabobank.riken.jp/data/RPMM0001/RawDataset/L_06_1.cdf¥"^^<http://ww
w.w3.org/2001/XMLSchema#anyURI>"
```

4.1 Using ABF* format files (*Reifycs's Analysis Base File)

```
res2 <- RPMM_get_rawdata_files(project = "RPMM0001", abf = TRUE)
head(res2$url)
## [1]
"¥"http://metabobank.riken.jp/data/RPMM0001/RawDataset/ABF/L_01_1.abf¥"^^<http
://www.w3.org/2001/XMLSchema#anyURI>"
## [2]
"¥"http://metabobank.riken.jp/data/RPMM0001/RawDataset/ABF/L_02_1.abf¥"^^<http
://www.w3.org/2001/XMLSchema#anyURI>"
## [3]
"¥"http://metabobank.riken.jp/data/RPMM0001/RawDataset/ABF/L_03_1.abf¥"^^<http
://www.w3.org/2001/XMLSchema#anyURI>"
```

```
## [4]
"¥"http://metabobank.riken.jp/data/RPMM0001/RawDataset/ABF/L_04_1.abf¥"^^<http
://www.w3.org/2001/XMLSchema#anyURI>"
## [5]
"¥"http://metabobank.riken.jp/data/RPMM0001/RawDataset/ABF/L_05_1.abf¥"^^<http
://www.w3.org/2001/XMLSchema#anyURI>"
## [6]
"¥"http://metabobank.riken.jp/data/RPMM0001/RawDataset/ABF/L_06_1.abf¥"^^<http
://www.w3.org/2001/XMLSchema#anyURI>"
```

Session information

```
## R version 4.1.0 (2021-05-18)
## Platform: x86_64-w64-mingw32/x64 (64-bit)
## Running under: Windows 10 x64 (build 19043)
##
## Matrix products: default
##
## locale:
## [1] LC_COLLATE=C                           LC_CTYPE=Japanese_Japan.932
## [3] LC_MONETARY=Japanese_Japan.932 LC_NUMERIC=C
## [5] LC_TIME=Japanese_Japan.932
##
## attached base packages:
## [1] stats     graphics  grDevices utils     datasets  methods   base
##
## other attached packages:
## [1] dplyr_1.0.7      plotly_4.9.4.1   ggplot2_3.3.5    rRPMM_0.99.0
## [5] SPARQL_1.16      RCurl_1.98-1.3   XML_3.99-0.6     BiocStyle_2.20.2
##
## loaded via a namespace (and not attached):
##  [1] tidyselect_1.1.1    xfun_0.24           bslib_0.2.5.1
##  [4] purrr_0.3.4         colorspace_2.0-2    vctrs_0.3.8
##  [7] generics_0.1.0      htmltools_0.5.1.1   viridisLite_0.4.0
## [10] yaml_2.2.1          utf8_1.2.1          rlang_0.4.11
## [13] jquerylib_0.1.4     pillar_1.6.1        glue_1.4.2
```

```
## [16] withr_2.4.2          DBI_1.1.1            lifecycle_1.0.0
## [19] stringr_1.4.0        munsell_0.5.0        gtable_0.3.0
## [22] htmlwidgets_1.5.3    evaluate_0.14        knitr_1.33
## [25] crosstalk_1.1.1      fansi_0.5.0          scales_1.1.1
## [28] BiocManager_1.30.16 jsonlite_1.7.2       digest_0.6.27
## [31] stringi_1.6.2        bookdown_0.22        grid_4.1.0
## [34] tools_4.1.0          bitops_1.0-7         magrittr_2.0.1
## [37] sass_0.4.0           lazyeval_0.2.2       tibble_3.1.2
## [40] crayon_1.4.1         tidyr_1.1.3          pkgconfig_2.0.3
## [43] ellipsis_0.3.2       data.table_1.14.0    assertthat_0.2.1
## [46] rmarkdown_2.9        httr_1.4.2           R6_2.5.0
## [49] compiler_4.1.0
```
