# Supplementary material for: Development of RIKEN Plant Metabolome MetaDatabase
Source: Plant Cell Physiol. 2021 Dec 17;63(3):433–40. doi: 10.1093/pcp/pcab173 (PMC8917833; doi:10.1093/pcp/pcab173)
Supplement: pcab173_Supp [file pcab173_supp.zip › pcp-2021-e-00297-File008.pdf]

Supplementary material 3. An example of a project report in *.Rmd file.

```
---
title: "RPMM_report"
author: "Atsushi Fukushima"
date: "`r Sys.Date()`"
output:
  BiocStyle::html_document:
package: rRPMM
vignette: |
  %\VignetteIndexEntry{rRPMM: test reports}
  %\VignetteEncoding{UTF-8}
  %\VignetteEngine{knitr::rmarkdown}
---

```{r setup, include = FALSE, message = FALSE}
knitr::opts_chunk$set(echo = TRUE)
```

RPMM: "RPMM0001"
```

This is a test report for
["RPMM0001"](http://metabobank.riken.jp/pmm/db/plantMetabolomics/http://metadb.riken.jp/db/plantMetabolomics/0.1/Project/RPMM0001) in RIKEN PMM.

````
```{r getData, message = FALSE}

library(SPARQL)

library(rRPMM)

res <- RPMM_get_project_info(projectid = "RPMM0001")

```
````

````
```{r showData, message = FALSE}

knitr::kable(data.frame(unlist(res)))

```
````

RPMM: "RPMM0003"

This is a test report for

["RPMM0003"](http://metabobank.riken.jp/pmm/db/plantMetabolomics/http://metadb.riken.jp/

db/plantMetabolomics/0.1/Project/RPMM0003) in RIKEN PMM.

````
```{r getData2, message = FALSE}

res2 <- RPMM_get_project_info(projectid = "RPMM0003")

```

```{r showData2, message = FALSE}

knitr::kable(data.frame(unlist(res2)))

```
````

RPMM_report

Atsushi Fukushima

2020-10-22

RPMM: “RPMM0001”

This is a test report for “RPMM0001” in RIKEN PMM.

```
source("RPMM_get_project_info.R")
library(SPARQL)
library(rRPMM)
res <- RPMM_get_project_info(projectid = "RPMM0001")
```

```
knitr::kable(data.frame(unlist(res)))
```

	unlist.res.
Project	http://metadb.riken.jp/db/plantMetabolomics/0.1/Project/RPMM0001
Title	“Unbiased characterization of genotype-dependent metabolic regulations by metabolomic approach in Arabidopsis thaliana”
TaxonID	http://purl.bioontology.org/ontology/NCBITAXON/3702
Species	“Arabidopsis thaliana”
PI_Name1	“KUSANO Miyako”
PI_Name2	“FUKUSHIMA Atsushi”
PI_Name3	“SAITO Kazuki”
PI_Affi_Name1	“RIKEN Center for Sustainable Resource Science”
PI_Affi_Name2	“Graduate School of Life and Environmental Sciences, University of Tsukuba”
PI_Affi_Name3	“RIKEN Plant Science Center”
PI_Affi_Name4	“Department of Molecular Biology and Biotechnology, Graduate School of Pharmaceutical Sciences, Chiba University”
Reference	http://rdf.ncbi.nlm.nih.gov/pubmed/18028551
Other_Information	http://www.ebi.ac.uk/metabolights/MTBLS40
Description	“Metabolites are not only the catalytic products of enzymatic reactions but also the active regulators or the ultimate phenotype of metabolic homeostasis in highly complex cellular processes. The modes of regulation at the metabolome level can be revealed by metabolic networks. We investigated the metabolic network between wild-type and 2 mutant (*methionine-over accumulation 1* [*mto1*] and *transparent testa4* [*tt4*]) plants regarding the alteration of metabolite accumulation in *Arabidopsis thaliana*. In the GC-TOF/MS analysis, we acquired quantitative information regarding over 170 metabolites, which has been analyzed by a novel score (ZMC, z-score of metabolite correlation) describing a characteristic metabolite in terms of
