# Supplementary material for: Development of RIKEN Plant Metabolome MetaDatabase
Source: Plant Cell Physiol. 2021 Dec 17;63(3):433–40. doi: 10.1093/pcp/pcab173 (PMC8917833; doi:10.1093/pcp/pcab173)
Supplement: pcab173_Supp [file pcab173_supp.zip › pcp-2021-e-00297-File009.pdf]

Supplementary material 4. An example of data reanalysis in RIKEN PMM using “rRPMM” (https://github.com/afukushima/rRPMM) and “eRah” (Domingo-Almenara et al. 2016) packages.

An example of data reanalysis in RIKEN PMM using rRPMM and eRah packages

Yusuke Aono[1] and Atsushi Fukushima[2]

[1] University of Tsukuba

[2] RIKEN Center for Sustainable Resource Science

2021-11-11

1 Introduction

To introduce our reanalysis approach, here, we share our GC-MS data reanalysis workflow using our rRPMM, an accessor for RIKEN Plant Metabolome Metadatabase (RIKEN PMM) and eRah package (Domingo-Almenara et al. 2016). This R-based workflow consists of the following steps: (1) obtaining all raw data (e.g., netCDF files) from a Project, (2) preprocessing the data (e.g., peak deconvolution and alignment), and (3) identifying and annotating metabolites with a public mass-spectral library such as GMD (Kopka et al. 2005). To demonstrate our workflow, we used publicly available GC-MS-based metabolome data from the root parasitic plant *Thesium chinense*, which were acquired using GC-TOF-MS (Ichihashi et al 2017), available from RIKEN PMM under the accession number “RPMM0026.”

2 Preparation

2.1 Downloading rawdata (netCDF files) using rRPMM package

An example R script for downloading netCDF files from RIKEN PMM is shown as follows:

```
library(SPARQL)
library(rRPMM)
library(stringr)

## get information from RIKEN PMM
res <- RPMM_get_rawdata_files(project = "RPMM0026")

## extract URL
urls<- lapply(res$url, function(x) {
  fileUrl <- as.character(str_extract(x[1], "http:.*cdf"))
})

## netCDF download
lapply(urls, function(x) {
  ## define file name
  destfile <- as.character(str_extract(x, "[^/]*cdf"))
  ## download
  download.file(x, destfile = destfile, method = "curl",
mode = wb)
  return( invisible() )

})
```

2.2 Placing netCDF files into folders

Before eRah analysis, netCDF files must be placed in different folders according to the sample class. In the dataset used for this example, two organs (the roots and

haustorium) were measured for two objects (lipophilic and polar metabolites). Accordingly, four class folders were created in a “rawdata” folder, and netCDF files were placed into these class folders.

```
working_directory/
+---rawdata/
    +---root_lipo/
    |    +---(netCDF files)
    +---root_polar/
    |    +---(netCDF files)
    +---haustorium_lipo/
    |    +---(netCDF files)
    +---haustorium_polar/
         +---(netCDF files)
```

In this document, the eRah analysis is performed using the folder above the “rawdata” folder as a working directory; the `createdt()` function creates two additional csv files in the “rawdata” folder (see the “Execution of eRah analysis” section). When executing this function, only the sample class folder can be placed under the “rawdata” folder.

2.3 Preparation of a mass-spectral library

This section describes the preparation of mass-spectral libraries. Libraries must be converted from the NIST Mass Search format (MSP) in advance. In this example, an MSP library available from RIKEN CompMS [“All records with Kovats RI (9062 unique compounds), GCMS DB-Public-KovatsRI-VS3.msp”] is converted for use in eRah. This library is written in the MSP format, but some text must be replaced before use in eRah. A descriptive example of the compounds in this library is shown below.

```
NAME: 1-NITROPYRENE; EI-B; MS

EXACTMASS: 247.0633285

FORMULA: C16H9NO2

SMILES: [O-1][N+1](=O)c(c4)c(c1)c(c3c4)c(c2cc3)c(ccc2)c1
```

```
ONTOLOGY: Pyrenes

INCHIKEY: ALRLPDGCPYIVHP-UHFFFAOYSA-N

RETENTIONTIME: -1

RETENTIONINDEX: 1872.217

QUANTMASS: 201

IONMODE: Positive

COLLISIONENERGY: 70eV

LICENSE: CC BY-SA

Comment: 

Num Peaks: 75

51  27

55  80

57  73

...
```

From the above items, replace the text as follows:

NAME -> Name

FORMULA -> Formula

RETENTIONINDEX -> RI

After replacement is complete, this library can be converted and saved using the following commands:

```
library(erah)
```

```
CompMS_public <- importMSP("GCMS DB-Public-KovatsRI-VS3.msp",
                              "CompMS_public",
                              "CompMS_public_20211019",
                              "All records with Fiehn RI (9062 unique compounds)")
save(CompMS_public, file = "CompMS_public.rda")
```

After executing the command, “CompMS_public.rda” will be created. This file can be read before executing compound annotation in eRah. Additionally, the GMD library from the GMD website can be used. For more information about each function, see the eRah manual.

3 Execution of eRah analysis

First, two csv files (“rawdata_inst.csv” and “rawdata_pheno.csv”) must be created using the `createdt()` function. This function refers to the path of the raw data storage folder (“rawdata” in this example) as a variable.

```
library(erah)
createdt("rawdata")
```

Here, a series of command examples up to data matrix acquisition are described. For more information about each function, see eRah manual.

```
## Deconvolution
ex <- newExp(instrumental = "rawdata/rawdata_inst.csv",
           phenotype = "rawdata/rawdata_pheno.csv",
           info = "rawdata Experiment")
ex.dec.par <- setDecPar(min.peak.width = 1,
                    avoid.processing.mz = c(35:69,73:75,147:149))
ex <- deconvolveComp(ex, ex.dec.par, down.sample = TRUE)
save(ex, file = "deconvolved.rda")

## Alignment
```

```
load("deconvolved.rda")
ex.al.par <- setAlPar(min.spectra.cor = 0.90,
                  max.time.dist = 3,
                  mz.range = 70:800)
ex <- alignComp(ex, alParameters = ex.al.par)

## missing compound recoverly
ex <- recMissComp(ex, min.samples = 3)

## Metabolite identification

load("CompMS_public.rda")
mslib <- CompMS_public

ex <- identifyComp(ex)
id.list <- idList(ex)
head(id.list[,1:4], n = 8)

## export to MSP
export2MSP(ex, export.id = NULL,
  id.database = mslib, store.path = getwd(),
  alg.version=1)

## export idlist
write.table(id.list, "idlist.txt", quote = FALSE, sep =
"¥t")

## export datalist
data.list <- dataList(ex, id.database = mslib, by.area =
TRUE)
write.table(data.list, "datalist.txt", quote = FALSE, sep =
"¥t")

save(list = ls(), file = ".Rdata")
```

4 References

GitHub - afukushima/rRPMM :https://github.com/afukushima/rRPMM
CRAN - Package erah: https://cran.r-project.org/web/packages/erah/index.html
Golm Metabolome Databese (GMD): http://gmd.mpimp-golm.mpg.de/
RIKEN CompMS website: http://prime.psc.riken.jp/compms/

5 Environment

The reanalysis of RIKEN PMM dataset described above was performed on a computer with Windows 10 Pro, 256 Gb of RAM, and a CPU AMD EPYC 7702P 64-Core Processor @ 2.00GHz.
